# AiZynthFinder 4.0: developments based on learnings from 3 years of industrial application

**DOI:** 10.1186/s13321-024-00860-x

**Published:** 2024-05-23

**Authors:** Lakshidaa Saigiridharan, Alan Kai Hassen, Helen Lai, Paula Torren-Peraire, Ola Engkvist, Samuel Genheden

**Affiliations:** 1 Molecular AI, Discovery Sciences, R&D, AstraZeneca, Gothenburg, Sweden; 2 Leiden Institute of Advanced Computer Science, Leiden University, Leiden, The Netherlands; 3 Molecular AI, Discovery Sciences, R&D, AstraZeneca, Cambridge, UK; 4 Institute of Structural Biology, Molecular Targets and Therapeutics Center, Helmholtz Zentrum München, Neuherberg, Germany

**Keywords:** Computer-aided synthesis planning, Retrosynthesis software, Multi-step retrosynthesis, Open-source

## Abstract

We present an updated overview of the AiZynthFinder package for retrosynthesis planning. Since the first version was released in 2020, we have added a substantial number of new features based on user feedback. Feature enhancements include policies for filter reactions, support for any one-step retrosynthesis model, a scoring framework and several additional search algorithms. To exemplify the typical use-cases of the software and highlight some learnings, we perform a large-scale analysis on several hundred thousand target molecules from diverse sources. This analysis looks at for instance route shape, stock usage and exploitation of reaction space, and points out strengths and weaknesses of our retrosynthesis approach. The software is released as open-source for educational purposes as well as to provide a reference implementation of the core algorithms for synthesis prediction. We hope that releasing the software as open-source will further facilitate innovation in developing novel methods for synthetic route prediction. AiZynthFinder is a fast, robust and extensible open-source software and can be downloaded from https://github.com/MolecularAI/aizynthfinder.

**Supplementary Information:**

The online version contains supplementary material available at 10.1186/s13321-024-00860-x.

## Introduction

Over the course of decades, the scientific community has grappled with the challenge of identifying the optimal sequence of chemical reaction steps capable of transforming a set of commercially available starting material into a desired chemical compound [1]. Solving this complex process entails searching through an extensive range of possible chemical transformations aimed at forming the target molecules. The increase in structural complexity of molecules poses further challenges by exponentially amplifying the time and effort required to explore solutions within a wide array of theoretically possible transformations [2]. The emergence of computer-aided synthesis planning (CASP) has greatly empowered chemists, serving as an invaluable tool in the realm of retrosynthetic planning [1]. At the core of this methodology lies the pioneering work of E. J. Corey, who formalized the process of retrosynthetic analysis, a method by which a target molecular compound is recursively decomposed into simpler, purchasable precursors [3].

Recent advancements in machine learning techniques, as well as the domain of deep neural networks and artificial intelligence (AI), have brought about substantial enhancements in predicting synthetic pathways, minimizing human intervention [4]. Two primary methodologies are commonly employed for neural network-guided one-step retrosynthesis to model the reverse reaction: template-based methods, and template-free methods [5]. In template-based retrosynthetic methods, a set of predefined molecular transformations are applied to the target molecule. These template rules are obtained either from experts as manually handwritten rules or by mining reaction databases [6]. The initial exploration into neural network guided template-based methods were pioneered by Segler and Waller [7]. The neural network predicts the most appropriate template to use based on a representation of the target molecule. On application of the predicted template, a set of precursors are generated and subsequently, this process is recursively employed to construct a retrosynthetic tree [8]. In the template-free methods, the reaction prediction task is often conceptualized as a sequence-to-sequence prediction problem. Here, the primary aim is to establish a mapping between a text sequence representing the reactants to a text sequence that represents the product, or conversely [9]. These text sequences can be achieved by using standardized notations like the Simplified Molecular-Input Line-Entry System (SMILES). The Molecular Transformer and the Chemformer are well-known template-free models that perform retrosynthesis as well as forward synthesis prediction [10–13]. For an extensive overview and classification of available retrosynthesis models, we recommend a recent review [14].

Numerous tools and platforms have been developed that offer retrosynthesis planning and other CASP solutions. Some of these tools are free for registered users like Chemical.AI [15] and IBM RXN [16], whereas other tools [17–22] are commercially available. A select few are entirely open source, including the AiZynthFinder tool from AstraZeneca [23], the ASKCOS suite of programs from MIT [1], LillyMol from Eli Lilly and Company [24], and Syntheseus from Microsoft [25]. We believe that open-source implementations would play a valuable role in advancing research within the field of computational chemistry. Therefore, we presented the AiZynthFinder tool in 2020 with the vision of contributing to scientific research and continuous development [23]. Apart from being used internally [26], the tool has seen a considerable uptake in the community, not at least shown by more than 200 citations (according to Google Scholar in May 2024). A few of the applications of AiZynthFinder is worth pointing out: a popular one has been to use retrosynthesis software output as ground-truth data for fast synthesizeability scores [27, 28]. Another use-case has been to use the single-step retrosynthesis model and combine it with biocatalysis models as in the RetroBioCat software [29]. Dolfus et al. used AiZynthFinder to generate routes that are then modified in a forward pass to generate compound libraries [30, 31]. Furthermore, it has been used to benchmark retrosynthesis algorithms [32, 33] and the output of AiZynthFinder can be read by LinChemIn [34] to facilitate comparison with other tools. On the more lightweighted side, a Twitter bot was integrated with AiZynthFinder to generate images of synthesis routes [35].

In this work, we describe the latest major release,  version 4.0, of AiZynthFinder. This new iteration incorporates substantial code improvements, novel features and expanded capabilities, all designed to address the evolving needs and challenges faced by the synthetic chemistry community, and particularly the medicinal chemists in AstraZeneca drug discovery projects. We provide descriptions of new features such as a policy to filter reactions during the tree search, additional expansion policies, multiple search algorithms, and route clustering and scoring functionalities. We will also provide an analysis of retrosynthesis experiments to illustrate typical use-cases of AiZynthFinder, offering an insight into the strengths and weaknesses of the tool. We will conclude by pointing out some outstanding challenges that we face when applying AiZynthFinder in drug discovery.

### Implementation

The AiZynthFinder is a Python-based platform, supporting Python 3.9 up to 3.11. In the tradition of open-source software development, we provide distribution of this new version along with all previous versions on GitHub under the MIT license [23, 36]. In addition to being available on GitHub, AiZynthFinder is also distributed through the Python Package Index (PyPI) [37], allowing convenient access and installation of the software. As the software is dependent on multiple free Python packages, dependency management has been facilitated using Poetry [38].

Before we offer insight into the newly implemented features and structure, we provide a concise overview of the previously implemented algorithm [23]: The retrosynthesis process is carried out by taking an input target molecule to decompose into purchasable precursors. The default search algorithm used is the Monte Carlo tree search (MCTS) [39] that together with a neural network-based policy is used to predict routes [40]. This is accomplished by iteratively expanding promising nodes in the tree search by applying reaction templates. As the tree reaches its maximum depth or if all molecules represented by a node are found in a given stock collection, a score is computed for the route and the resulting precursors. This process is iteratively repeated until it reaches the maximum number of iterations, or a specified time limit [23]. The AiZynthFinder retains this as its foundational algorithm, with an introduction to multiple features aimed at enhancing the flexibility of the retrosynthesis process.

**Fig. 1:**
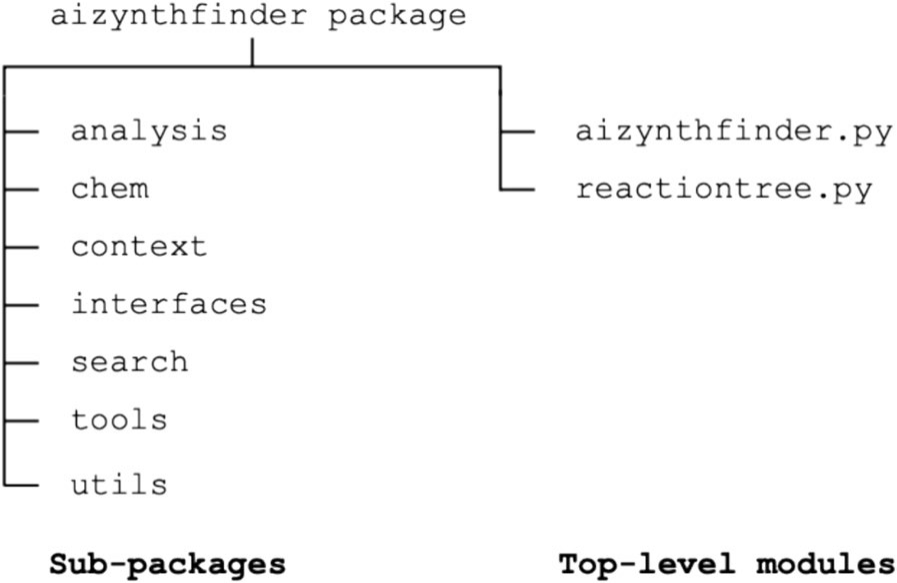
The AiZynthFinder python package structure, outlining top-level modules and sub-packages

Since 2020, significant research and implementation have been focused on introducing and expanding new features aimed at enhancing the software. We provide a concise overview of the main features introduced during this period, alongside the revised structure of the package that can be seen in Fig. 1. The functionalities within the sub-packages play an integral role in the overall execution of the algorithm by the top-level modules. The chem sub-package is responsible for managing molecules and reactions, using RDKit [41] routines. Functionalities pertaining to configuration input, filter and expansion strategies, scoring and stocks are provided within the context sub-package. The search sub-package holds the tools for employing different search algorithms beyond MCTS on the target molecule, while the analysis sub-package handles the analysis of the tree search results and efficient management of collections of synthetic routes. The tools and utils sub-packages provide general tools and functionalities applicable across all sub-packages, including data downloading, logging, file management, and more. The reactiontree module combines some of these features to construct a reaction tree representing a single synthetic route. All these sub-packages collectively contribute to the functionality of the aizynthfinder module, enabling the complete retrosynthesis process. This retrosynthesis process can be executed through user interfaces like the graphical user interface (GUI) and command-line interface (CLI), using functions and routines provided in the interfaces sub-package. The sub-package training from the previous structure of the AiZynthFinder package [23] has been moved to AiZynthTrain [42] for building and training expansion models.

### Context: filter and expansion policies, scoring and stock

The sub-package context comprises three key sub-package—policy, scoring and stock. The policy package contains two different functionalities, a filter policy to remove unrealistic reactions and an expansion policy to suggest new reactions. The default filter policy, as proposed by Segler et al. [7], utilizes a trained neural network model that classifies reactions as being feasible or infeasible. Any infeasible reactions are immediately removed from the tree search. Additionally, the codebase is adaptable to facilitate the integration of additional filter strategies. For instance, based on a user suggestion we added a filter that removes expansions where the number of reactants does not match what is expected from the template. The context sub-package also includes expansion policy mechanisms, whereby the functionality encompasses the use of expansion strategies to generate chemical transformations from a given target molecule, expanding it into simple precursor molecules. Notably, two available expansion mechanisms used in the retrosynthesis process are the template-based expansion and the SMILES-based (or template-free) expansion. In the template-based expansion, a trained neural network is used for recommending the most probable templates for application to the target molecule. This process yields a sorted list of the most probable reaction templates that can be applied, along with their corresponding probabilities [23].

Additionally, we have introduced the ModelZoo [43, 44] package that can be downloaded from Github (https://github.com/PTorrenPeraire/modelsmatter_modelzoo) as a plug-in to our software, offering users the flexibility to employ any expansion strategies, whereby the SMILES representation of the target molecule is broken down into simple precursors. This feature offers the possibility of applying the most suitable contemporary single-step retrosynthesis model complementing the multi-step retrosynthetic process. The ModelZoo currently supports models such as the Chemformer [12, 13], MHNreact [45] and LocalRetro [46]. Furthermore, we have implemented the functionality of incorporating multiple expansion strategies simultaneously. This mechanism provides the option to either obtain a consolidated list of highly probable reaction templates and their associated probabilities obtained from all the provided expansion strategies or solely from the first strategy listed. For instance, one can combine the general retrosynthesis model with the Ringbreaker model [47], or augment predictions with look-up from reaction databases.

The construction of the expansion and filter models using the TensorFlow [48] framework has led to a significant dependence on TensorFlow for both training and inference within AiZynthFinder, affecting the software’s overall run-time. To address this, we have implemented a measure to use converted template-based models in the ONNX [49] format for inference, effectively moving the TensorFlow dependency to the training phase. The utilization of ONNX models for inference has yielded significant improvements in the start-up time, average search time for a solution and a minor impact on the total number of solved solutions. In the Supporting Information, we detail a comparison of Tensorflow and ONNX performance that shows that the start-up time with ONNX was found to be approximately 2.4 times faster than TensorFlow, while the search time for a solution using ONNX was found to be approximately 1.7 times faster. Apart from offering speed-ups, moving to use ONNX as the ML back-end for the template-based models allows us to be model-agnostic, applying template-based models trained by other groups in AiZynthFinder. For instance, we converted the PyTorch-based model trained by Chen et al. [50] that has been used in several publications [40] and compared it to our UPSTO-based model [8] (see Supporting Information for further information).

The scoring package holds a collection of scoring functions that can be applied to the retrosynthesis process to score MCTS nodes or synthetic routes, further enhancing the algorithm with a strategy for building and selecting optimal routes. The default scorer calculates scores for a node, or a reaction route, based on the respective maximum tree depth as well as the fraction of starting material in stock. Additionally, the module offers alternative scoring methods to score nodes and reaction routes by considering factors such as the fraction of starting material available in stock, the number of reactions required to reach a specific node in the tree, the count of precursors in a node or route, the average occurrence of templates to reach a specific node, the cost of molecules and reactions as proposed by Badowski et al. [51], and many more.

The stock package holds the mechanism by which the retrosynthesis search is terminated because a set of purchasable building blocks is reached. The default stock is an in-memory set of InChI keys of the available material, but we have since AiZynthFinder version 1.0 implemented additional stopping criteria such as minimum amount and maximum price allowed for building blocks. We also recently implemented the possibility to use the MolBloom package [52] as stock, which reduces the memory consumption of AiZynthFinder significantly. A benchmarking of this functionality is detailed in Supporting Information.

### Search algorithms

The sub-package search includes the implementation of the MCTS search algorithm including notable enhancements to its overall functionality. These improvements include a mechanism to prevent the formation of cycles when expanding the search tree. We have also implemented features that do not change the underlying algorithm but make the utilization of expensive models such as Chemformer more effective, including sibling node-expansion and model caching [12]. Moreover, we have expanded the search capabilities by incorporating additional search algorithms like the Breadth-First Search, Depth First Proof Number Search [53], and Retro* [50], within the search sub-package. These search algorithms are based on AND/OR-trees compared to the super-node representation used in MCTS [54].

### Interfaces: AiZynthFinder and AiZynthExpander, GUI and CLI

The aizynthfinder.py module serves as the primary interface to the retrosynthesis process, containing core functionalities encapsulated within the AiZynthFinder and AiZynthExpander classes. The AiZynthFinder class contains the main tree search loop, using functionalities from the chem and search sub-packages to build synthesis routes. The AiZynthExpander class integrates functionalities from chem, context and reactiontree to execute single-step retrosynthesis. By combining the functionalities of both these classes, the complete multi-step retrosynthesis process is formed.

**Fig. 2:**
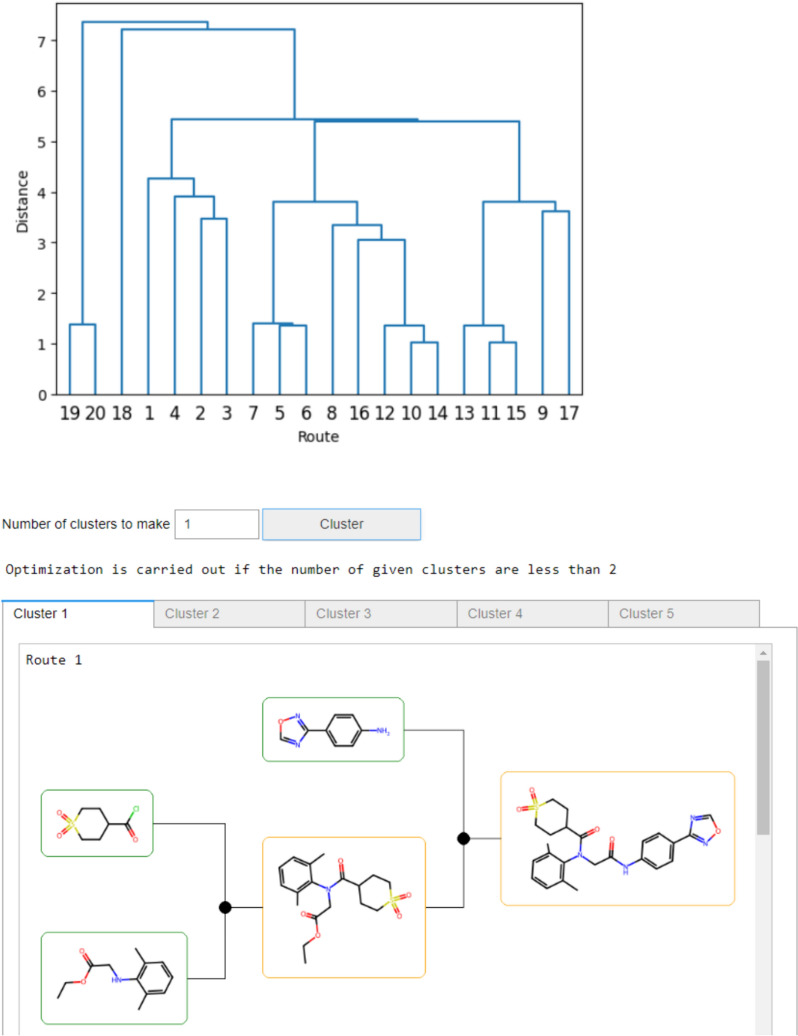
Jupyter GUI for AiZynthFinder highlighting the route clustering. The relationship of the 20 routes extracted from the search of the Amenamevir drug is shown in a dendrogram. The bottom-part of the GUI shows a tab for each of the five clusters obtained when optimizing for the number of clusters. Each tab shows a pictorial representation of the routes

The end-users can access these functionalities through two interfaces—the command-line interface (CLI) and the graphical user interface (GUI), whose functionalities reside within the interfaces sub-package. The GUI offers capabilities to execute tree search on single compounds directly within a Jupyter [55] notebook. This interface also provides users with the possibilities to perform route analysis and clustering of routes. An example is shown in Fig. 2 for the drug Amenamevir [56]. The route clustering is obtained from a tree edit distance computation as previously outlined [57]. In contrast, the CLI allows users to perform tree search on batches of compounds. Users can submit batches of compounds and obtain comprehensive results for the provided SMILES. Additionally, the CLI provides a checkpoint mechanism, enabling users to track processed compounds in case of a process restart.

## Results and discussion

To illustrate the usage of AiZynthFinder, we performed several retrosynthesis experiments as outlined in Table 1 (details can be found in Supporting Information). We compiled sets of compounds typical for how AiZynthFinder is used, and augmented them with compound sets from open sources. For in-house compounds, we selected one set of approximately 65,000 compounds that were designed by chemists and a set of approximately 112,000 compounds generated by the de novo design platform REINVENT [58] for ten different drug projects. For the publicly available compounds, we selected 100,000 compounds from the ChEMBL [59] and GDB MedChem databases [60], respectively. We will now present an analysis of these retrosynthesis experiments from numerous perspectives.

**Table 1:** Summary of retrosynthesis experiments

Target compounds	Number of targets	Models trained on	Stock collection
AZ designs	65,300	Reaxys + Pistachio + ELN	AstraZeneca internal
Reinvent	112,600	Reaxys + Pistachio + ELN	AstraZeneca internal
ChEMBL	100,000	USPTO	E-molecules + ZINC
GDB	100,000	USPTO	E-molecules + ZINC

The number of targets for which we find at least one route where all the starting material is in stock is 80% and 86% for the AZ designs and Reinvent sets, respectively (see Table 2). For ChEMBL, we only find solutions to 71% of the targets, somewhat lower than the AstraZeneca sets. However, this could be explained by the extended number of iterations used for the AstraZeneca sets as recommended in a recent study of search hyperparameters [61]. For the GDB set we only find solutions to about 10%, highlighting a disconnection of the current template-based model trained on historical reaction data, with the chemistry needed to find synthesis routes for the enumerated, and therefore potentially non-synthesizable, GDB compounds. The median search time is about 40 s for the ChEMBL set and 90 s for the Reinvent sets, i.e. it is likely that one would obtain a retrosynthesis route within two minutes. The number of routes found is above 100 for all sets, although the number of solved routes is less than 100. Finally, the routes generated for the Reinvent compounds are generally the longest, most convergent and require the most starting materials. AZ designs require slightly shorter routes and slightly less starting materials. For the public target sets, GDB require slightly longer routes than ChEMBL, although the amount of starting materials is comparable. We also performed retrosynthesis analysis on the ChEMBL compounds using the 1.0 release of AiZynthFinder and the USPTO-based expansion model available in 2020 [8] (see Table S2). This setup could solve approximately two percent less targets than the current setup, confirming the previously made observation that for USPTO-based models there is not a large difference between the previous model of Thakkar et al. and the current re-trained model [42]. The median search time has decreased considerably with the 4.0 version mainly due to the use of ONNX, as described above. This comparison shows that for gross metrics like the one presented in Table 2, the quantitative performance of retrosynthesis has improved only slightly compared to the earlier version. However, the additional features added to the code base like filter policies and algorithmic improves the quality of the proposed routes, which is not directly reflected in Table 2.

**Table 2:** General statistics of the retrosynthesis experiments

Target set	% solved targets	Median search time (s)	No. of routes^a^	No. of routes^b^	No. of solved routes	Average no. of starting material	Average no. of steps	Average longest linear sequence
AZ designs	80.30	82.34	155.72	187.43	65.45	4.31	4.40	3.67
Reinvent	85.61	87.34	148.61	170.04	71.54	5.07	6.04	4.80
ChEMBL	70.96	37.03	121.80	200.21	38.45	2.67	1.97	1.85
GDB	10.12	47.19	145.50	184.01	17.88	2.93	2.98	2.87

^a^Only for targets for which no solved routes were found

^b^Only for targets for which at least one solved route was found

**Fig. 3:**
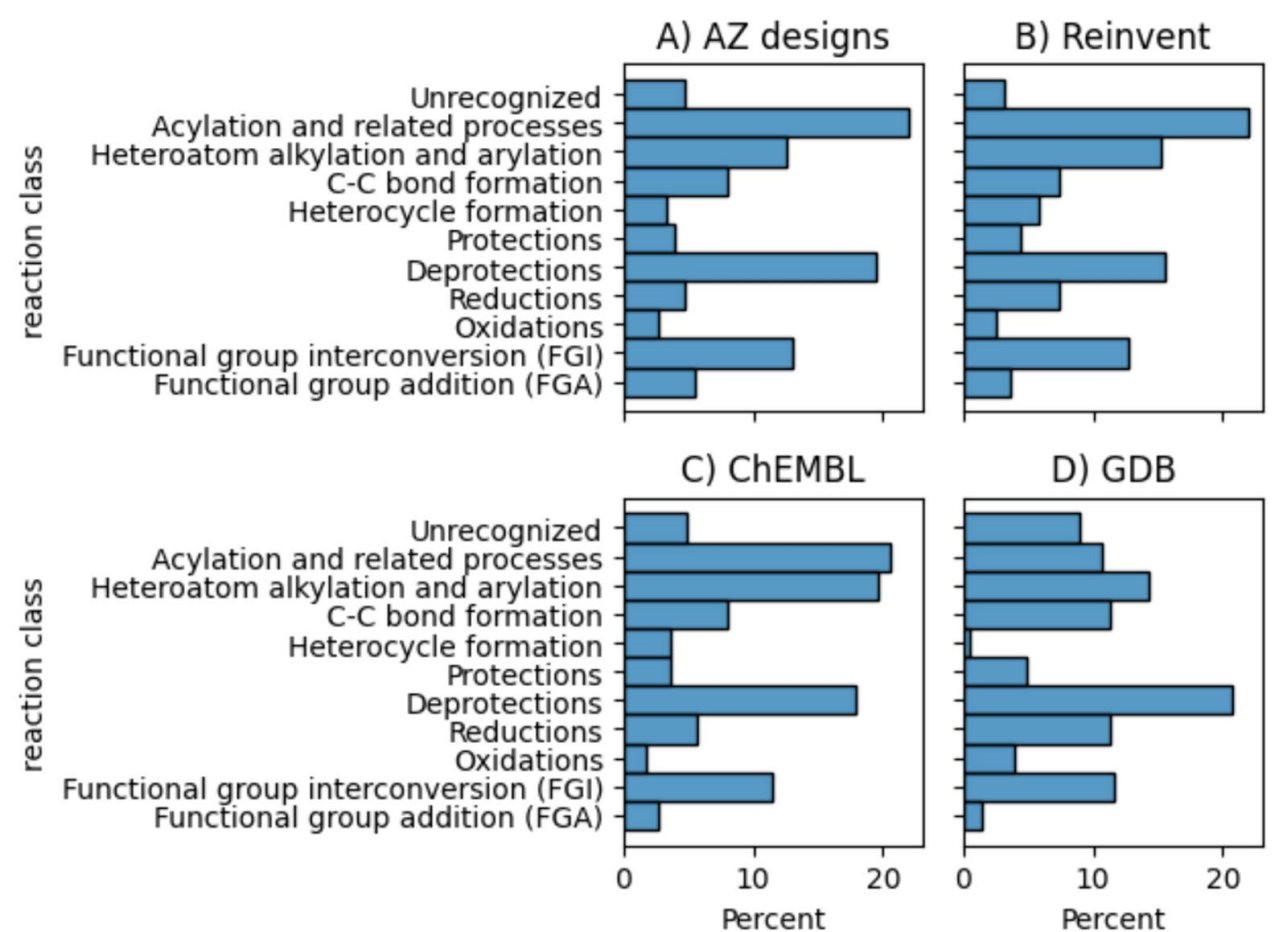
The distribution of different reaction classes in the synthesis routes predicted for the different target sets

Next, we analysed the classification of the reactions used in the synthesis routes. For the AZ designs, Reinvent, and ChEMBL sets, the three most commonly used reactions are acylations, alkylation/alyrations, and deprotections (see Fig. 3). On the contrary, oxidations, heterocycle formation and protections are rarely used. It is notable that the usage of deprotections is not countered by a usage of protections. One possible reason could, of course, be that the starting material contains protection groups, necessitating the need for deprotection. However, in our experience, the routes predicted by AiZynthFinder often contain sub-optimal (de-)protection strategies, probably because these are relatively abundant reaction classes leading them to be suggested by the retrosynthesis model. The distribution of reaction classes for the GDB target sets is slightly different than for the other three classes. There is very little heterocycle formations used and fewer acylations, whereas the number of unrecognized reactions is increased.

**Fig. 4:**
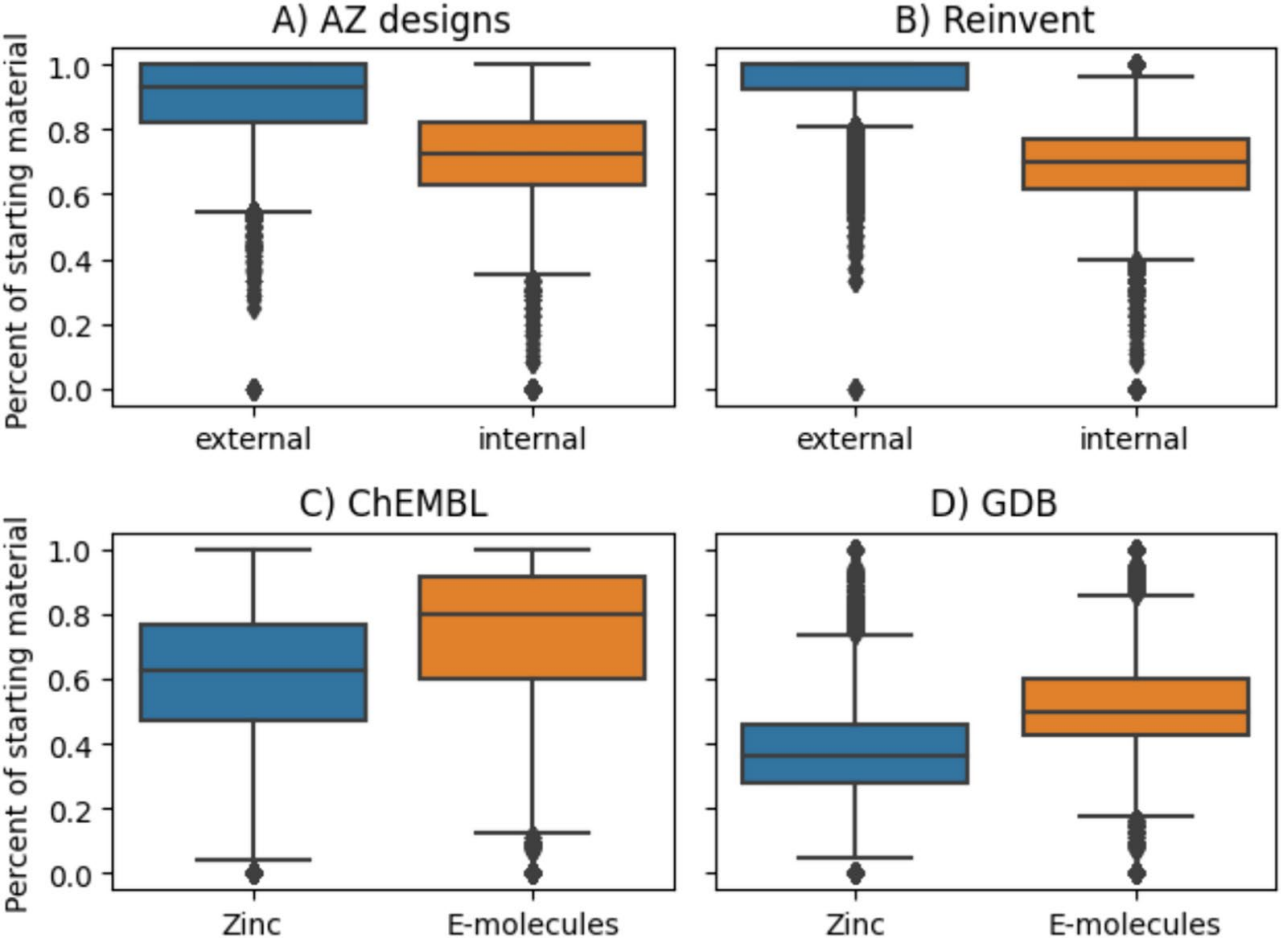
The percentage of starting materials found in external or internal stocks for the AZ designs and Reinvent target sets, or ZINC and E-Molecules stocks for the ChEMBL and GDB target sets

Next, we analyze the impact of different stock sets by contrasting the origin of used starting materials that are part of found synthesis routes when different stock sets are combined during the search. For the AZ designs and Reinvent compound sets, we used a stock set that is a combination of external vendors and building blocks available at AstraZeneca storages. When analyzing the proportion of starting material that was found in either the external or the internal stocks, we see that on average, the starting material is most likely found in the external stocks, but the internal stock covers on average 70% of the starting material (see Fig. 4). In order to improve the lead time for synthesis, one could do an analysis of the most frequently used externally available building blocks and make sure that they are available at AstraZeneca’s internal storages. For the ChEMBL and GDB target sets, we used a combination of stocks that we created for the first release of AiZynthFinder from the ZINC database, and the E-Molecules building blocks, a popular choice in multi-step retrosynthesis publications [40, 50]. We see in Fig. 4 that in general, the E-Molecules stock set is most useful as it covers on average 80% of the starting material, whereas ZINC stock only covers 60% on average. However, as there is virtually no compute overhead in using more than one stock in AiZynthFinder, one can argue that using both ZINC and E-Molecules is preferable.

**Fig. 5:**
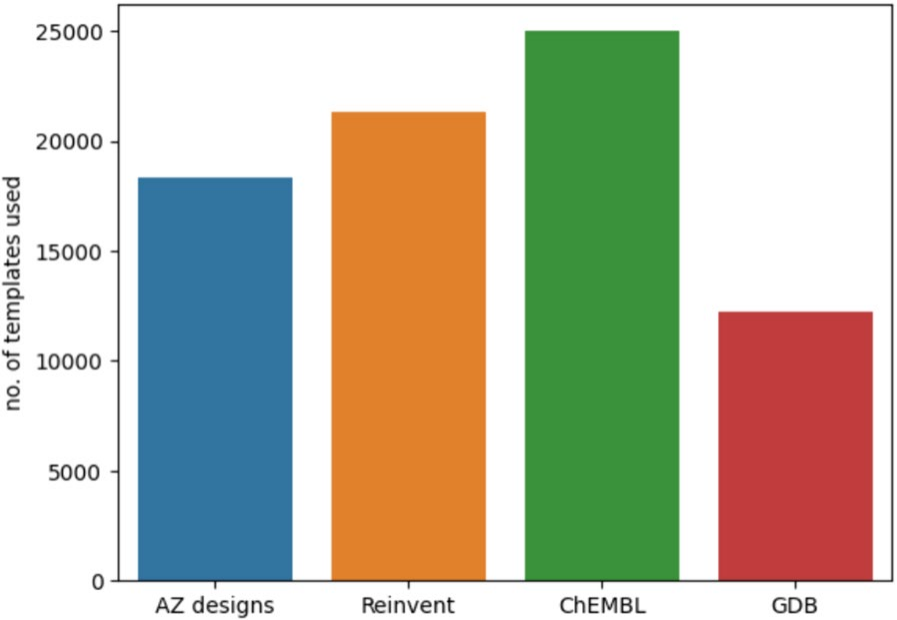
The number of unique templates used in the routes for different target sets. For AZ designs and Reinvent the model trained on Reaxys, Pistachio and AstraZeneca ELN is used and for ChEMBL and GDB the USPTO-based model is used (see Table 1)

There are about 180,000 templates in the internal AstraZeneca expansion model, and about 45,500 templates in the public USPTO-based expansion model, extracted by an automatic procedure [41]. An interesting question is how many of these templates are used to predict routes for the target sets. In Fig. 5, we show that between 12,000 (for GDB) and 25,000 (for ChEMBL) templates are used when deploying the USPTO-based expansion model. This implies that for ChEMBL, about 59% of the USPTO-based templates are used, but for AZ designs, only 10% of the templates derived from Reaxys, Pistachio and AstraZeneca ELNs are used. Thus, we can conclude that either there is an enormous challenge in prioritizing templates or that a majority of the templates extracted are redundant. Most likely, it is a combination of both.

**Fig. 6:**
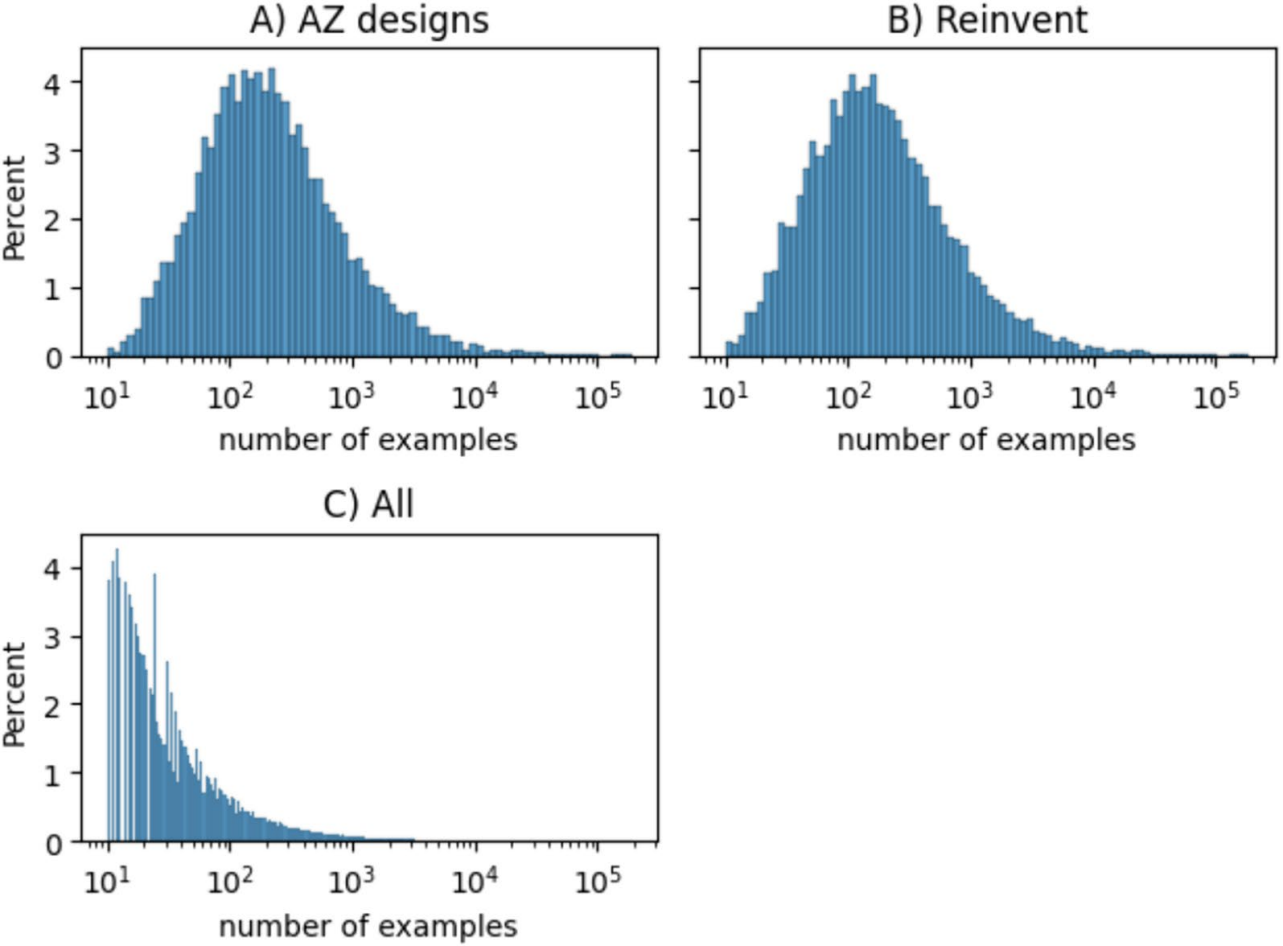
The distribution of the number of reaction examples per template for **A** the templates used in the routes for the AZ designs, **B** the templates used in the routes for the Reinvent targets, and **C** all templates in the internal AZ model. The y-axis indicates the percentage of the total number of templates

To investigate the potential prioritization issue, we plotted the distribution of the number of reaction examples that were used to derive a template. For the set of all templates, we see the typical skewed distribution of the number of reactions: most of the templates have very few examples, and very few have more than 10,000 reactions (see Fig. 6C). However, if we look at the distribution for the templates used in the predictions for the AZ designs and Reinvent targets, we see a normal distribution, centred on a bit more than 100 examples, and a long tail towards higher number of examples. Thus, we are unlikely to utilize templates with few examples. A similar shift in the distribution is shown in an analysis for the ChEMBL and GDB target sets, but to a much smaller degree (see Figure S1). In the internal expansion model, we have set the cut-off at ten examples, whereas for the USPTO-based model, it is set to three examples. This analysis shows that perhaps we could increase the cut-off, considering that the internal reaction dataset is about 10 ten times larger than the USPTO dataset, making a cut-off at 30 examples could be a reasonable target to focus on the used, most common reaction templates. Alternatively, we could investigate a few-shot model [45] or an approach for reducing the number of templates based on graph subsets [32].

## Conclusions and outlook

AiZynthFinder is used daily in-house, and chemists can choose to run their own retrosynthesis experiments or analyse the results of one of the automatically submitted jobs that is triggered for every designed compound. Many of the features that we have described in this text were driven by business needs, to improve the accuracy and speed of the retrosynthesis engine. However, despite some progress made, it is still inherently difficult to compare output from retrosynthesis experiments on a large scale [25, 54], and improvements are often judged on a case-by-case basis.

Although a substantial number of new features have been introduced, there are a number of limitations of the current approach. AiZynthFinder and other retrosynthesis software suffer from some severe limitations in efficient usage of the one-step retrosynthesis models. Those models are inherently trained for batch inference, whereas the multi-step algorithms operate inherently on single compounds. We have implemented features to our MCTS algorithm to alleviate this, but there is still much improvement possible. Furthermore, we also showed herein that AiZynthFinder is incapable of taking advantage of the broad chemical space that is encoded in the template-based model (see Fig. 5). A second challenge is the balancing of multiple expansion models, something that has been implemented in AiZynthFinder. In production, we typically use three expansion models in parallel: the general template-based model, the RingBreaker, and a reaction look-up function. Due to that the priors from these models does not operate on the same scale, we have, for instance, observed an overuse of the RingBreaker model. A third challenge is the accurate scoring of routes, which is essential for the software to recommend routes rather than serving as an ideation tool. A more robust scoring could both better guide the tree search and aid in selecting the best routes.

AiZynthFinder will continue to incorporate solutions to these challenges as well as other innovations, and continue being an essential tool for retrosynthesis analysis, for both industry and academia.

### Supplementary Information

Supplementary material 1.

## Declarations

### Availability and requirements

Project name: AiZynthFinder.

Project home page: http://www.github.com/MolecularAI/aizynthfinder

Operating system(s): Platform independent.

Programming language: Python 3.

Other requirements: several open source python packages.

License: MIT.

Any restrictions to use by non-academics: none.
