# Supplementary material for: AiZynthFinder 4.0: developments based on learnings from 3 years of industrial application
Source: J Cheminform. 2024 May 23;16:57. doi: 10.1186/s13321-024-00860-x (PMC11112899; doi:10.1186/s13321-024-00860-x)
Supplement: Supplementary file 1 — Supplementary material 1. [file 13321_2024_860_MOESM1_ESM.docx]

# Supporting information:

# AiZynthFinder 4.0: developments and learnings from three years of industrial application

Lakshidaa Saigiridharan, Alan Kai Hassen, Helen Lai, Paula Torren-Peraire, Ola Engkvist, Samuel Genheden

**Details of retrosynthesis experiments in Table 1**

We use filter models derived from USPTO or Reaxys to match the expansion policy as detailed previously [62]. The AstraZeneca stock is a 5M stock comprised of both external sources and building blocks available in AstraZeneca storage. The ZINC stock was created by us for the first release of AiZynthFinder. The E-Molecule stock was created by downloading a flat file from the E-Molecules homepage (https://downloads.emolecules.com/free/) in January 2023. The training of the expansion models was carried out with AiZynthTrain. The experiments with the ChEMBL and GDB targets were carried out with a maximum depth of 7, whereas the experiments with the AZ Designs and REINVENT targets were carried out with a maximum depth of 12. We extracted between 10 and 50 routes sorted by the score driving the MCTS [8] (a combination of the maximum depth of the route and the fraction of starting material in stock).


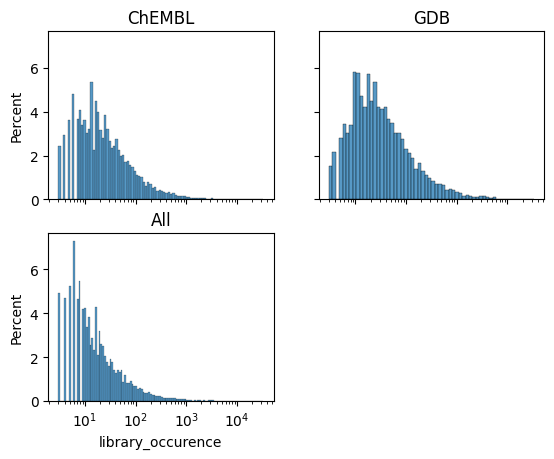


**Figure S1** – The distribution of the number of reaction examples per template for A) the templates used in the routes for the ChEMBL compounds, B) the templates used in the routes for the GDB targets, and C) all templates in the USPTO model.

**ONNX and Tensorflow comparison**

We performed retrosynthesis experiments on 100,000 compounds from the ChEMBL database, using internal filter and expansion models, as well as AstraZeneca internal stock. All other settings were default ones. The main results are shown in Table S1 and indicate that ONNX outperformed TensorFlow in this exercise. The start-up time with ONNX was found to be approximately 2.4 times faster than TensorFlow, while the search time for a solution using ONNX was found to be approximately 1.7 times faster.

**Table S1 – Benchmarking of time comparison between ONNX and TensorFlow**

|  | ONNX | TF |
| --- | --- | --- |
| Average start-up time (s) | 30.24 | 73.16 |
| Total number of solved targets | 81738 | 81598 |
| Average search time per target (s) | 38.17 | 65.95 |

**AiZynthFinder 1.0 experiments**

We installed AiZynthFinder 1.0 in according to the instructions on Github and downloaded the USPTO-based expansion model available in 2020. We then performed retrosynthesis experiments on the 100K ChEMBL compounds with the E-Molecules stock. Note that a filter-policy was not used as this was not supported by the 1.0 version of the software, In order to complete the performance on all compounds, we had to add a try-except-clause at one place in the code-base, but otherwise the experiments could be completed effortlessly. Some of the analyses could however not be performed with the 1.0 version due to lack of functionality. The comparable statistics of the experiments are shown in Table S2.

**Retro* retrosynthesis model**

We downloaded the Retro* template-based retrosynthesis model following the instructions on GitHub (<https://github.com/binghong-ml/retro_star>). We converted the PyTorch model to ONNX format and the templates to a format required by AiZynthFinder. We also computed a hash [63] for each template to be able to compare the template space models trained with the AiZynthTrain package. We then ran experiments on 100 ChEMBL compounds using the filter model derived from USPTO data and a stock that was a combination of ZINC and E-Molecules. It is then possible to compare it to the experiments that we performed using the USPTO-based model derived with the AiZynthTrain package.

There are approximately 381K templates in the Retro* model compared to the approximately 43K templates in the USPTO model derived with AiZynthTrain. So, there are plenty of templates that are only in the Retro* model (see Figure S2).


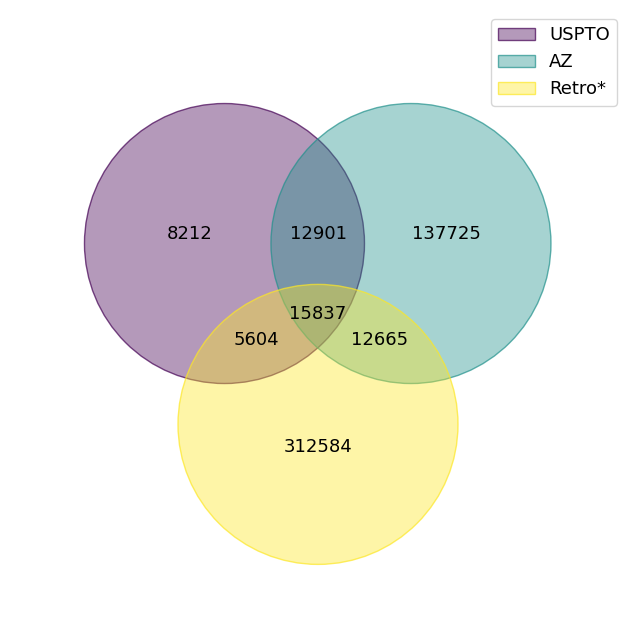


**Figure S2 –** Template overlap between USPTO (derived with AiZynthTrain), AstraZeneca model (AZ), and Retro* (also derived from USPTO but with another procedure).

The comparison between the models is shown in Table S3 (the USPTO row is the same in Table 2 in the main text). The performance is similar between the two models, although the Retro* model results in slightly lower number of solved targets and takes considerably longer time. The experiments using Retro* utilize more templates in the absolute sense, but only about 15% of the total number of templates are used compared to 60% for the model derived with AiZynthTrain.

**Table S2 – Comparison of the additional retrosynthesis experiments on the ChEMBL compound set**

| Model | % solved targets | Median search time | No. of routes^b^ | No. of solved routes | Average no. of starting material | Average no. of steps | Average longest linear sequence | No. Of used templates |
| --- | --- | --- | --- | --- | --- | --- | --- | --- |
| USPTO | 70.96 | 37.03 | 121.80 | 38.45 | 2.67 | 1.97 | 1.85 | 24988 (59%) |
| USPTO-2020^a^ | 69.15 | 50.37 | N/A | N/A | 2.45 | 1.93 | 1.80 | N/A |
| Retro* | 68.07 | 67.91 | 108.96 | 32.94 | 2.69 | 2.04 | 1.95 | 59260 (15%) |

^a^ Computed with version 1.0 of AiZynthFinder. N/A values indicate that these number could not be computed in a comparable way due to lack of functionality.

^b^ Only for targets for which no solved routes were found

**MolBloom filters as stocks**

We performed retrosynthesis experiments on 5,000 compounds from the ChEMBL database using internal filter and expansion models, and all other settings were default ones. As stock, we used an AstraZeneca stock of 5M compounds, or 25M compounds from E-Molecules. The stocks were represented as either an in-memory set of InChI keys or as a MolBloom filter. The MolBloom filters were either 10 or 100MB large, and created from either canonicalized SMILES or InChI keys. We used the in-memory set of InChI keys as the ground-truth and compared to this to the MolBloom flagging of the starting materials of the routes extracted from retrosynthesis experiments. The interesting results are the sum of the true positive (TP) and true negative (TN) predictions as well as the balanced accuracy for three levels of comparison: 1) the molecule level, i.e. if using MolBloom could “indicate” if the molecule was in stock or not, 2) the route level, i.e. if all the starting material in a route is in stock or not, and 3) the target level, i.e. if at least one route was solved. The results are shown in Table S3. A few observations can be made: first, we see that the accuracy at all levels is increased with filter size, as expected. Second, we see that InChI key representation outperform the SMILES representation. Using a 100 MB filter and InCHI key representation we can achieve a 100% accuracy at the target level for both the AstraZeneca and E-Molecules stocks.

**Table S3 – Accuracy of MolBloom filter as a stock**

| Stock set | Filter size | Representation | Validation level | Accuracy | TP+TN |
| --- | --- | --- | --- | --- | --- |
| AstraZeneca | 10 MB | SMILES | Leaves | 99.1% | 99.6% |
|  |  |  | Routes | 98.5% | 98.5% |
|  |  |  | Targets | 93.1% | 95.9% |
| AstraZeneca | 100 MB | SMILES | Leaves | 99.9% | 99.7% |
|  |  |  | Routes | 99.2% | 98.9% |
|  |  |  | Targets | 98.6% | 97.7% |
| AstraZeneca | 10 MB | InChI keys | Leaves | 99.4% | 99.9% |
|  |  |  | Routes | 99.4% | 99.6% |
|  |  |  | Targets | 93.7% | 97.9% |
| AstraZeneca | 100 MB | InChI keys | Leaves | 99.99% | 99.99% |
|  |  |  | Routes | 99.99% | 99.9% |
|  |  |  | Targets | 100.0% | 100.0% |
| E-Molecules | 100 MB | InChI keys | Leaves | 99.99% | 99.9% |
|  |  |  | Routes | 99.97% | 99.97% |
|  |  |  | Targets | 100.0% | 100.0% |
